# Short-term effects of different PM2.5 ranges on daily all-cause mortality in Jinan, China

**DOI:** 10.1038/s41598-022-09057-4

**Published:** 2022-04-05

**Authors:** Zhixiang Ma, Xiangwei Meng, Cai Chen, Baoting Chao, Chuanzhen Zhang, Wei Li

**Affiliations:** 1 Biomedical Engineering Institute, School of Control Science and Engineering, Shandong University, Jinan, 250016 Shandong China; 2 Department of Gastroenterology, The First Affiliated Hospital of Shandong First Medical University & Shandong Provincial Qianfoshan Hospital, Jingshi Road 16766#, Jinan, 250014 China; 3 Shandong Institute of Advanced Technology Chinese Academy of Sciences, Jinan, 250000 China; 4 Department of Radiology, Shandong Provincial Hospital Affiliated to Shandong First Medical University, Jinan, 250021 Shandong China

**Keywords:** Environmental sciences, Medical research, Risk factors

## Abstract

To examine the effects of different PM_2.5_ concentration ranges on daily all-cause mortality, 8768 all-cause deaths were recorded in the database of the Shandong Provincial Hospital Affiliated to Shandong First Medical University. Data of air pollutants (PM_2.5_ and O_3_) concentration were provided by the Jinan Environment Monitoring Center. The relative risk of all-cause mortality was assessed using a quasi-Poisson regression model after adjusting for confounding factors. The concentrations of PM2.5 were divided into four ranges 0–35 μg/m^3^; 35–75 μg/m^3^; 75–115 μg/m^3^; 115–150 μg/m^3^. There was no significant relationship between PM2.5 exposure and all-cause deaths in individuals aged < 60 years. However, for individuals aged ≥ 60 years, there was a significant positive association between exposure concentrations and all-cause deaths within the ranges 0–35 μg/m^3^, 35–75 μg/m^3^, and 115–150 μg/m^3^ with a mortality increase of 1.07 (1.01, 1.13), 1.03 (1.00, 1.05), and 1.05 (1.01, 1.08), respectively. When the population aged ≥ 60 years was stratified into gender groups, exposure to PM2.5 in the range 0–35 μg/m^3^ increased the mortality risk in men but not women. All-cause mortality in women, but not men, increased significantly with exposure to PM2.5 in the ranges of 35–75, 75–115, and 115–150 μg/m^3^.

## Introduction

Particulate matter in the air refers to the dispersed solid, liquid or solid–liquid suspended body in the air. Particulate matter in the air is divided into coarse particulate matter PM_10_ (aerodynamic diameter below 10 μm), fine particulate matter PM_2.5_ (aerodynamic diameter below 2.5 μm), and ultrafine particles (aerodynamic diameter less than 0.1 μm). The toxicity and pathogenicity of particulate matter are closely related to its diameter composition and source. PM_2.5_ is mainly caused by the burning of fossil fuels such as oil, coal or wood. The particles produced by power plants, industrial production, residential heating and motor vehicle driving are usually composed of carbon, transition metals complex organic molecules, sulfate and nitrate. Soluble components (ultrafine particulate matter) of PM_2.5_ can enter the blood circulation through the alveolar capillaries, while insoluble components can be deposited in the lungs, obstruct airflow and affect the respiratory system^1–3^.

O_3_ is formed by photochemical reactions of oxidants and hydrocarbons in the atmosphere under the catalysis of sunlight. As a major component of acid rain and photochemical smog, the strong oxidation of O_3_ can cause serious damage to cell walls and have acute effects on the lungs and respiratory system. There is ample evidence that ground-level ozone impairs lung function and stimulates the respiratory system^3–5^. Exposure to ozone (and the pollutants that produce it) is significantly associated with premature death, asthma, bronchitis, heart attacks and other heart and lung problems.

The adverse effects of airborne particulate matter ≤ 2.5 µm, PM_2.5_ on public health, especially in the respiratory and cardiovascular systems, have been studied for nearly half a century. The formation of PM_2.5_ and its adverse impact on public health are evident in both developed and developing countries^6–8^. Various studies in Europe, the United States, and developing countries such as China, India, and Korea found that entire populations were affected by short-term exposure to fine particulate matter and that there was a positive correlation between PM_2.5_ levels and mortality^9–14^. In addition, substantial epidemiological evidence demonstrates that ground-level fine particulate matter is linked to various respiratory diseases, including asthma, chronic obstructive pulmonary disease, lung cancer^15–17^, and cardiovascular mortality^18–20^.

However, the results of all-cause mortality associated with exposure to PM_2.5_ are inconsistent; therefore, public awareness of the risk of this type of exposure is low^21–23^. Moreover, few studies to date investigated the PM_2.5_ ranges that poses no health risk. For this reason, a recommended PM_2.5_ concentration is needed to minimize the adverse health effects^24^.

The objective of this study is to examine the effects of different PM_2.5_ ranges on all-cause mortality and provide public health recommendations to avoid exposure to PM_2.5_.

## Materials and methods

### Data source

Daily concentrations of PM_2.5_ in 24-h intervals and ozone (O_3_) in 1-h intervals averaged in urban areas of Jinan, China, from 2013 to 2015, were obtained from 14 permanent air quality monitoring stations of Jinan Environmental Protection Bureau. PM_2.5_ is monitored by Beta attenuation monitoring technique, light scattering, and micro oscillatory balance method, O_3_ is monitored by spectrophotometry, ultraviolet spectrophotometry, and chemiluminescence method, at each air quality monitoring stations under supervision of Jinan Environmental Protection Bureau. Also, the use of monitors follows the *Technical specifications for operation and quality control of ambient air quality automated monitoring system for particulate matter.* Daily mean air temperatures and relative humidity in the corresponding period were provided by the Jinan Bureau of Meteorology. We use expectation maximization to make up for the missing values.

Data on the daily mortality of the registered population of Jinan for the period 2013–2015 were recorded in the database of Shandong Provincial Hospital Affiliated to Shandong First Medical University. Detailed demographic information, including age, gender, date of hospital admission, date of hospital discharge, admission diagnosis, discharge diagnosis codes, and current residence. Mortality data on total non-accidental causes (codes A00–R99), cardiovascular disease (codes I00–I99), and respiratory disease (codes J00–J98) were classified according to International Classification of Diseases Tenth Revision 10 (ICD-10). The data on all-cause mortality were stratified by gender (male and female) and age (< 60 and ≥ 60 years).

### Data analysis

PM_2.5_ concentrations were classified into four ranges: 0–35 μg/m^3^, 35–75 μg/m^3^, 75–115 μg/m^3^ and 115–150 μg/m^3^—based on the Chinese new air quality index (AQI) (GB3095-2012) released by the Ministry of Environmental Protection (MEP). To establish the four PM2.5 concentration ranges, we set all concentrations outside the range as “NA”.

A quasi-Poisson regression model with natural splines was used to assess the impact of different PM_2.5_ ranges on daily all-cause mortality because the daily death counts in Jinan approximately followed a Poisson distribution. This regression model is used to adjust inference for overdispersion^25^. The natural cubic spline for mean temperatures with 5 degrees of freedom and relative air humidity with 3 degrees of freedom (*df*) was controlled to analyze all-cause mortality based on Akaike’s Information Criterion (AIC) for lag effects of up to 3 days^26^. Confounding factors such as day of the week and holidays were included as dummy variables.

The natural cubic spline smoothing function degree of freedom for mean temperature and relative air humidity is determined as follows:

$$\begin{array}{*{20}l} Log[E(Yt)] = \alpha + ns(Temp,df) + \beta_{1} factor(DOW) + \beta {}_{{2}}factor(Holiday) \hfill \\ Log[E(Yt)] = \alpha + ns(RH,df) + \beta_{1} factor(DOW) + \beta {}_{{2}}factor(Holiday). \hfill \\ \end{array}$$

*Yt* represents the death counts on day t. *E(Yt)* represents the expected death counts on day t, *ns* stands for the natural cubic spline smoothing function, *Temp* represents the mean temperature, *RH* represents the relative air humidity, *DOW* and *Holiday* stands for the day of the week effect and legal holidays respectively, β_1_ and β_2_ are the coefficient of *DOW* and *Holiday* respectively. The degree of freedom of the mean temperature factor is N (N = 2,3,…,6). Obtain the magnitude of the corresponding AIC of the equation when N is different, and the minimum value of AIC is the optimal degree of freedom.

Different PM_2.5_ ranges were added into the above basic model to establish a single-pollutant model. Multi-pollutant models with PM_2.5_ and O3, with multi-day moving average lag structures [from a lag of 0 to 1 day (mean) to a lag of 0 to 3 days (mean)], were used for sensitivity analysis to determine the stability of the model.

The relative risk (RR) and corresponding 95% confidence interval (CI) for an increase of 10 μg/m^3^ in pollutant concentration were estimated to assess the impact of different PM_2.5_ ranges on daily counts of all-cause mortality. P-values smaller than 0.05 were considered statistically significant.

Stratified analyses of exposure to different PM_2.5_ ranges based on gender (male or female) and age (< 60 years and ≥ 60 years) were performed to find associations with daily all-cause mortality.

## Results

### Distribution of ambient pollutants and weather data

**Figure 1:**
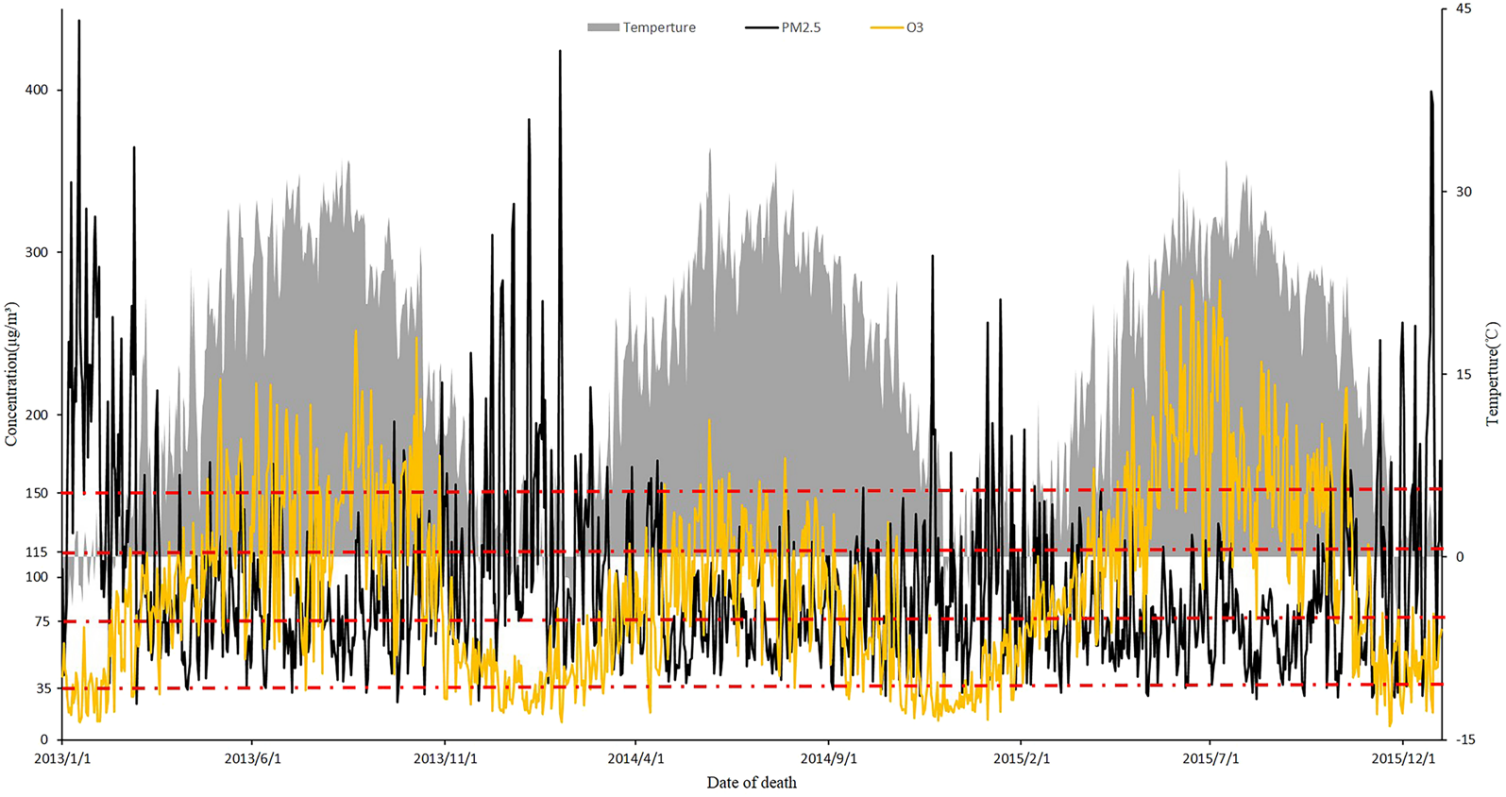
Distribution of daily ambient pollutant concentrations and temperature in Jinan, China, from 2013 to 2015.

The mean daily concentrations of PM_2.5_ and O_3_ from 2013 to 2015 were 96 μg/m^3^ and 102.4 μg/m^3^, and these values are 1.28- and 0.64-fold higher than those reported by the new Chinese ambient air quality standards (GB3095-2013). The levels of PM_2.5_ in 625 of 1095 days exceeded the annual secondary national 24-h ambient air quality standards (75 μg/m^3^). The frequency distribution of daily ambient pollutant levels and temperatures are shown in Fig. 1.

### Data description

A total of 8768 all-cause deaths (5462 men and 3306 women) for the period 2013–2015 were recorded in the database of Shandong Provincial Hospital Affiliated to Shandong First Medical University. The percentage of individuals aged < 60 and ≥ 60 years was 38.79% (3401/8768) and 61.21% (5367/8768), respectively. The distributions of the daily concentrations of air pollutants, weather parameters, and deaths are shown in Table 1.

**Table 1:** Daily distribution of air pollutant levels, weather parameters, and deaths in Jinan, China, from 2013 to 2015.

Variable	Mean and SD	Min	P25	P50	P75	Max	IQR
**Pollutants**							
PM_2.5_ (μg/m^3^)	96 ± 58	22	59	82	116	443	57
O_3_ (μg/m^3^)	96 ± 57	8	48	86	134	283	87
**Meteorological data**							
Temperature (°C)	15.2 ± 10.3	− 9.4	5.8	16.6	24.1	33.7	18.3
Relative air humidity (%)	56 ± 20	15	41	55	70	100	29
**Daily deaths**							
From all causes	8 ± 3	1	4	6	10	15	20
**Gender**							
Male	5 ± 1	0	1	4	6	8	11
Female	3 ± 1	0	1	3	4	6	12
**Age**							
< 60	3 ± 1	0	1	3	5	6	3
≥ 60	5 ± 1	1	1	5	7	11	19

According to MEP, air quality was good (green category) in 4.11% of the days, moderate (yellow category) in 38.36% of the days, poor for sensitive groups (orange category) in 32.24% of the days, poor (red category) in 12.42% of the days, and very poor (purple category) in 12.88% of the days for all populations. PM_25_ concentration and air quality index values in the study period are shown in Table 2.

**Table 2:** PM_2.5_ levels and air quality index values in Jiang, China, from 2013 to 2015.

PM_2.5_ levels (μg/m^3^)	2013		2014		2015		Air quality index values	MEP air quality	Category
	N	(%)	N	(%)	N	(%)			
≤ 35	12	3.3	9	2.5	24	6.6	≤ 50	Good	Green
36–75	116	31.8	150	41.1	154	42.2	51–100	Moderate	Yellow
76–115	119	32.6	125	34.2	109	29.9	101–150	Poor for sensitive groups	Orange
116–150	48	13.1	44	12.1	44	12.0	151–200	Poor	Red
> 150	70	19.2	37	10.1	34	9.3	> 200	Very poor	Purple

### Daily all-cause mortality

For individuals aged ≥ 60 years, there were strong associations between exposures on lag days 0, 1, 2, and 3 and means of lags 0–1, 0–2 and 0–3 to the three PM_2.5_ concentrations ranges 0–35, 35–75, and 115–150 μg/m^3^. The statistically significant relative risks (RR) with 95% confidence intervals (CI) for daily all-cause mortality from exposure to the three PM_2.5_ ranges were 1.07 (1.01, 1.13), (lag 1, 0–35 μg/m^3^), 1.03 (1.00, 1.05), (lag 0, 35–75 μg/m^3^) and 1.05 (1.01, 1.08), (lag 0, 115–150 μg/m^3^). For a moving average lag structure of 01 the statistically significant relative risks RR (95% CI) for daily all-cause mortality from exposure to PM_2.5_ in the ranges of 0–35, 35–75, and 115–150 μg/m^3^ were 1.10 (1.02, 1.18), 1.04 (1.01, 1.07), and 1.06 (1.02, 1.11) respectively. Furthermore, RR (95% CI) for daily all-cause mortality from exposure to 115–150 μg/m^3^ of PM_2.5_ was 1.06 (1.01, 1.11) in lag 02 (Table 3).

**Table 3:** Relative risk (RR) with 95% confidence interval (CI) for daily All-cause mortality from exposure to different PM_2.5_ ranges in Jinan, China, from 2013 to 2015, both sexes, all ages.

All-cause	0–35 μg/m^3^ [RR, (95% CI)]	35–75 μg/m^3^ [RR, (95% CI)]	75–115 μg/m^3^ [RR, (95% CI)]	115–150 μg/m^3^ [RR, (95% CI)]
Lag 0	1.03 (0.98–1.09)	1.03 (1.00–1.05)*	1.02 (0.99–1.05)	1.05 (1.01–1.08)*
Lag 1	1.07 (1.01–1.13)*	1.02 (1.00–1.04)	1.01 (0.99–1.04)	1.03 (1.00–1.07)
Lag 2	0.95 (0.91–1.00)	0.99 (0.97–1.01)	1.00 (0.97–1.02)	1.00 (0.97–1.04)
Lag 3	0.96 (0.92–1.01)	0.97 (0.95–0.99)	0.98 (0.96–1.01)	0.99 (0.96–1.03)
Lag 01	1.10 (1.02–1.18)*	1.04 (1.01–1.07)*	1.03 (1.00–1.07)	1.06 (1.02–1.11)*
Lag 02	1.04 (0.95–1.14)	1.03 (0.99–1.06)	1.02 (0.98–1.07)	1.06 (1.01–1.11)*
Lag 03	1.00 (0.91–1.11)	1.00 (0.96–1.04)	1.01 (0.96–1.06)	1.05 (0.99–1.11)

*p < 0.05.

**Figure 2:**
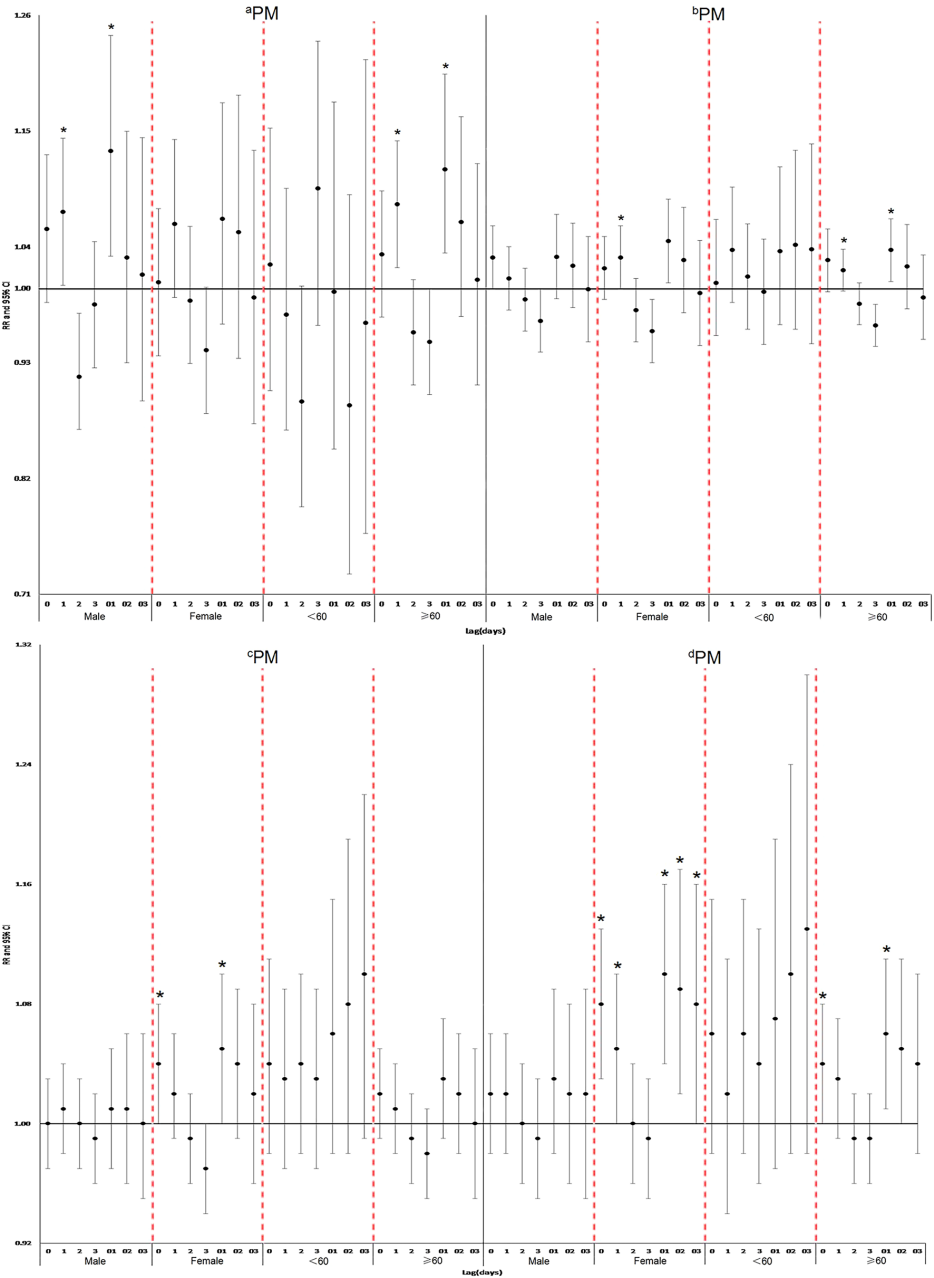
Lag structures of age and gender-specific relative risk (RR) of daily mortality from exposure to different PM_2.5_ ranges. ^a^0–35 μg/m^3^, ^b^35–75 μg/m^3^, ^c^75–115 μg/m^3^, ^d^115–150 μg/m^3^. *p < 0.05.

Stratified analysis based on gender and age indicated that there was a significant relationship between all-cause mortality and a PM_2.5_ range of 0–35 μg/m^3^ in men in lags 1 and 01. All-cause deaths in women significantly increased with exposure to PM_2.5_ in the ranges of 35–75 μg/m^3^, 75–115 μg/m^3^, and 115–150 μg/m^3^ in lag 1; lags 0 and 01; and lags 0, 1, 01, 02, and 03, respectively. There were no significant associations between PM_2.5_ exposure and all-cause mortality in individuals aged < 60 years. All-cause deaths in individuals aged ≥ 60 years were significantly correlated with exposure to ranges of 35–75 μg/m^3^, 75–115 μg/m^3^, and 115–150 μg/m^3^ in lags 1 and 01; lags 1 and 01; and lags 0 and 01, respectively (Fig. 2).

**Figure 3:**
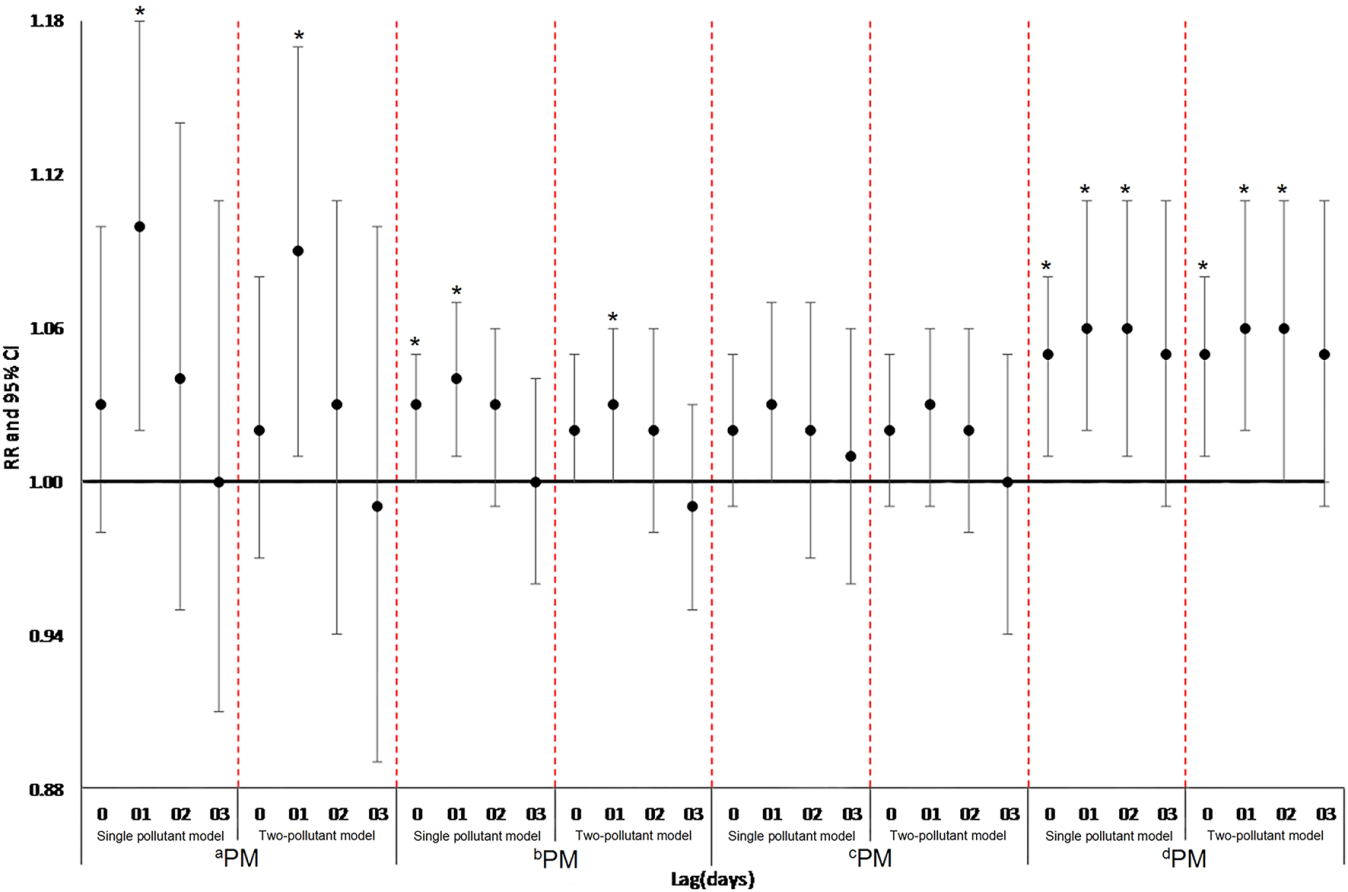
Lag structures of relative risk (RR) and 95% confidence interval (CI) between single pollutant models and two-pollutant models for different PM_2.5_ ranges in lag 0 to lag 03. ^a^0–35 μg/m^3^, ^b^35–75 μg/m^3^, ^c^75–115 μg/m^3^, ^d^115–150 μg/m^3^. *p < 0.05.

The results of sensitivity analysis indicated that the relative risk at different PM_2.5_ ranges for daily all-cause mortality generally decreased slightly after including O3 in the multi-day moving average lag structures (Fig. 3). This may be related to the strong collinearity between different Pollutants. Some studies also believe that dual-pollutant models will increase the standard deviation of model fitting, so the significance of statistical analysis is weak^27^.

## Discussion

To our knowledge, this epidemiologic study is the first to examine the association of PM_2.5_ concentration ranges with all-cause mortality in Asia. The results indicated that, except for the PM_2.5_ range of 75–115 μg/m^3^, the concentrations of PM_2.5_ in the ranges 0–35 μg/m^3^, 35–75 μg/m^3^, and 115–150 μg/m^3^ were significantly associated with mortality from all causes for individuals aged ≥ 60 years. At the optimum lag structure, the statistically significant relative risks RR (95% CI) for daily all-cause mortality from exposure in the ranges 0–35 μg/m^3^, 35–75 μg/m^3^, and 115–150 μg/m^3^ of PM_2.5_ were 1.10 (1.02, 1.18), 1.04 (1.01, 1.07), and 1.06 (1.02, 1.11) respectively. This indicated that the adverse impacts on public health do not decrease as pollutant levels decrease. The statistically significant relative risks RR (95% CI) for daily all-cause mortality from exposure in the range 0–35 μg/m^3^ of PM_2.5_ were 1.10 (1.02, 1.18). This means that in the concentration range of 0 to 35 μg/m^3^, the effects of PM_2.5_ were stronger as the concentration increased. In the same way, in the concentration ranges of 35 to 75 μg/m^3^ and 115 to 150 μg/m^3^, we can get the same conclusion. . In addition, as shown in Table 3, in the PM_2.5_ concentration range of 0 to 35 μg/m^3^, RR (95% CI) for daily all-cause mortality was 1.10 (1.02, 1.18), and Fig. 2 indicated that there was a significant relationship between all-cause mortality and a PM_2.5_ range 0–35 μg/m^3^ in men and individuals aged ≥ 60 years in lags 1 and 01, both of these indicated that consistent with other studies^28–30^, even for concentrations lower than 35 μg/m^3^, PM_2.5_ is a significant risk factor for all-cause mortality.

For individuals aged ≥ 60 years, the association between all-cause deaths and PM_2.5_ exposures was statistically significant at ranges of 0–35 μg/m^3^, 35–75 μg/m^3^, and 115–150 μg/m^3^. The lack of significance in the 75–115 μg/m^3^ range may be because of the relatively fewer deaths in this range. Furthermore, the daily temperatures corresponding to concentrations of 75–115 μg/m^3^ were higher than those at 0–35, 35–75, and 115–150 μg/m^3^. The impact of different PM_2.5_ ranges on mortality may be due to differences temperatures^31,32^.

The results of a previous study on the gender-specific effects of particulate matter were inconsistent^33^. The results of the gender-stratified analysis demonstrated that female subjects were more sensitive to the PM_2.5_ in the ranges of 35–75 μg/m^3^, 75–115 μg/m^3^, and 115–150 μg/m^3^, whereas male subjects were more sensitive to PM_2.5_ in the range of 0–35 μg/m^3^, indicating that men are more susceptible to lower PM_2.5_ concentrations than women. Smoking is a critical environmental risk factor, and one study suggested that the estimated impact of air pollution might be stronger in nonsmokers than smokers^34^. A potential reason for this difference may be that women have slightly stronger airway reactivity and smaller airways than men^35^. Moreover, the adverse impacts of additional exposure to PM_2.5_ may be overcome by the oxidative and inflammatory effects of smoking^36^.

Older individuals had increased susceptibility to PM_2.5_ ranges of 35–75 μg/m^3^, 75–115 μg/m^3^, and 115–150 μg/m^3^ compared with younger individuals, possibly because the former group has a weaker immune system and higher sensitivity to these particles^37,38^. However, there was no significant association between PM_2.5_ exposure and all-cause mortality in individuals aged < 60 years, indicating that the general population should avoid high levels of PM_2.5_ (≥ 75 μg/m^3^).

This study has some limitations. First, the study selected the mean air pollutant concentration from each monitoring site in Jinan as the exposure concentration; nonetheless, individual exposure may depend on other factors, including the type of outdoor activity, physical fitness, and living habits, potentially causing exposure measurement errors or underestimating the impact of air pollution. In addition, this study belongs to the field of ecological research, and the conclusions cannot prove causality but merely indicate the relationship between air pollutants and all-cause mortality.

## Conclusions

There was no significant relationship between PM_2.5_ exposure and all-cause deaths in individuals aged < 60 years. However, for individuals aged ≥ 60 years, there was a significant positive association between exposure concentrations and all-cause deaths within the ranges 0–35 μg/m^3^, 35–75 μg/m^3^, and 115–150 μg/m^3^ with a mortality increase of 1.07 (1.01, 1.13), 1.03 (1.00, 1.05), and 1.05 (1.01, 1.08), respectively. When the population aged ≥ 60 years was divided into gender groups, exposure to PM2.5 in the range 0–35 μg/m^3^ increased the mortality risk in men but not women. All-cause mortality in women, but not men, increased significantly with exposure to PM2.5 in the ranges of 35–75, 75–115, and 115–150 μg/m^3^.
